# Lipid Droplet Accumulation and Impaired Fat Efflux in Polarized Hepatic Cells: Consequences of Ethanol Metabolism

**DOI:** 10.1155/2012/978136

**Published:** 2012-03-11

**Authors:** Benita L. McVicker, Karuna Rasineni, Dean J. Tuma, Mark A. McNiven, Carol A. Casey

**Affiliations:** ^1^Liver Study Unit-Research Service (151), VA Nebraska-Western Iowa Health Care System, 4101 Woolworth Avenue, Omaha, NE 68105, USA; ^2^Departments of Internal Medicine and Biochemistry and Molecular Biology, University of Nebraska Medical Center, Omaha, NE 68198, USA; ^3^Department of Biochemistry and Molecular Biology, Mayo Clinic College of Medicine, Rochester, MN 55905, USA

## Abstract

Steatosis, an early manifestation in alcoholic liver disease, is associated with the accumulation of hepatocellular lipid droplets (LDs). However, the role ethanol metabolism has in LD formation and turnover remains undefined. Here, we assessed LD dynamics following ethanol and oleic acid treatment to ethanol-metabolizing WIF-B cells (a hybrid of human fibroblasts (WI 38) and Fao rat hepatoma cells). An OA dose-dependent increase in triglyceride and stained lipids was identified which doubled (*P* < 0.05) in the presence of ethanol. This effect was blunted with the inclusion of an alcohol metabolism inhibitor. The ethanol/ OA combination also induced adipophilin, LD coat protein involved in the attenuation of lipolysis. Additionally, ethanol treatment resulted in a significant reduction in lipid efflux. These data demonstrate that the metabolism of ethanol in hepatic cells is related to LD accumulation, impaired fat efflux, and enhancements in LD-associated proteins. These alterations in LD dynamics may contribute to ethanol-mediated defects in hepatocellular LD regulation and the formation of steatosis.

## 1. Introduction

Alcohol abuse and alcoholic liver disease (ALD) are major health problems both in the USA and worldwide. The most prevalent manifestations of ALD are the presence of fatty liver (hepatic steatosis), alcoholic hepatitis, and cirrhosis. Of these manifestations, it is noted that hepatic steatosis is a reversible early stage of ALD whose presence has been related to the liver's enhanced sensitivity to damaging triggers such as oxidative stress and endotoxins [1, 2]. Thus, the aberrant content of lipids in hepatocytes can act as a key “first hit” in the progression of ALD making lipid accumulation a prime target for therapeutic intervention. Remarkably, little is known about the regulatory mechanisms involved in the accumulation of intracellular lipids which are stored in dynamic organelles called lipid droplets (LDs). Furthermore, it is unclear how LD formation, degradation (lipolysis), or export is affected, particularly in the hepatocyte by the adverse effects of alcohol exposure and the metabolism of ethanol.

The organelle identified as having a central role in the accumulation of lipids in hepatocytes is the LD. LDs are intracellular stores of neutral lipids, predominately cholesterol esters, and triglycerides that are bound by a phospholipid monolayer [3]. Long considered to be inert, LDs have recently attracted great interest as dynamic structures at the hub of lipid and energy metabolism. In general, LDs are thought to originate from the endoplasmic reticulum from where they are trafficked through the cytoplasm, interacting with various organelles and transporting lipids as the energy needs of the cell dictate [4, 5]. In the healthy liver, LDs in hepatocytes play a crucial role in the packaging and distribution of lipids as lipoproteins [6]. However, in disease states such as ALD, the accumulation of LDs (hepatic steatosis) is likely due to disruption in those packaging and distribution roles as alcohol exposure induces impairments in LD formation, degradation, and/or export processes. Ultimately, excessive LD accumulation occurs in hepatocytes which can lead to lipotoxicity with consequences of inflammation and subsequent cell death. However, little is known about how ethanol exposure/metabolism alters the regulation of LD accumulation and degradative processes in the hepatocyte.

The liver and, to a lesser extent, the gastrointestinal tract are the main sites of alcohol metabolism. Within the liver, there are two main pathways of alcohol metabolism, alcohol dehydrogenase (ADH) and cytochrome P-450 2E1 (CYP2E1) [7–9]. Relating alcohol metabolism and generated toxic products (i.e., acetaldehyde) to mechanisms by which ethanol causes fatty liver appears to be complex. Historically, it has been proposed that reducing equivalents generated during ethanol oxidation inhibit steps of the tricarboxylic acid cycle and oxidation, thereby inhibiting fatty acid oxidation [10, 11]. Another proposed mechanism involved in the development of alcoholic fatty liver is enhanced lipogenesis with support of several studies demonstrating significant increases observed in hepatic lipogenesis following chronic ethanol administration [12–14]. Alternatively, it has been shown that the persistence of fatty liver may involve the inhibition of lipoprotein export mechanisms, possibly via formation of acetaldehyde protein adducts and associated alterations to the microtubule network in the cell [15, 16]. Thus, the production of toxic metabolites of ethanol is thought to play a significant role in altering the trafficking and utilization of LDs in hepatocytes. Indeed, it now appears that similar to previously identified ethanol-mediated impairments to hepatocellular receptors, ligands, and endocytic processes [17–19], LDs may be regulated by interactions involving classical trafficking pathways and are therefore highly susceptible to damaging alterations induced by ethanol metabolites. To better define the role of ethanol metabolism in hepatic steatosis, we analyzed LD accumulation and impaired fat efflux in hepatoma cell cultures (WIF-B cells), a well-established *in vitro* model for studying the consequences of ethanol metabolism on hepatocellular trafficking events.

WIF-B cells are differentiated cells of hepatic origin that are a hybrid clone of human fibroblasts (WI38) crossed with rat hepatoma cells [20]. WIF-B cells exhibit long-term viability in culture, develop a hepatocellular-polarized phenotype, and express human genes coding for liver-specific proteins (e.g., albumin and fibrinogen) [21, 22]. The WIF-B cells have been shown to adequately mimic *in vivo* hepatocellular functions such as polarity, protein secretion, and transport [23–25]. Additionally, our laboratory has demonstrated that WIF-B cells are an ideal *in vitro* model for studying the effect of ethanol on cellular processes as the cells were found to exhibit ADH and CYP2E1 activities allowing for the efficient metabolism of ethanol [26]. In our previous work, we characterized several ethanol-mediated cellular defects in the alcohol-treated WIF-B cells which linked ethanol metabolism to apoptotic-inducing pathways and with the potential involvement of altered targeting of proteins [27]. In this study, alcohol-treated WIF-B cells were used to characterize the role ethanol metabolism has in the generation, accumulation, efflux, and lipolysis of LDs.

## 2. Materials and Methods

### 2.1. Materials

F-12 Coon's modified culture medium, 4-methylpyrazole (4MP), Oil Red O (ORO), oleic acid (OA), and fatty acid free bovine serum albumin (BSA) were obtained from Sigma Chemical Co. (St. Louis, MO). Heat-inactivated fetalplex was obtained from Gemini Bio-Products (Woodland, CA). BODIPY 493/503 was purchased from Invitrogen (Carlsbad, California). Buffered formalin and isopropanol were obtained from Fisher Scientific (Pittsburgh, PA). UltraCruz mounting media containing 4,6-diamidino-2-phenylindole (DAPI) was obtained from Santa Cruz Biotechnology, Inc (Santa Cruz, CA). All other materials were reagent grade.

### 2.2. WIF-B Cell Culture and Treatment

WIF-B cells were cultured in F-12 media containing 3.5% heat-inactivated fetalplex in a 7% CO_2_ atmosphere as described previously [27]. Briefly, the cells were seeded on sterilized glass coverslips or directly in tissue culture dishes and cultured for 6 days to obtain a maximal-polarized phenotype prior to the various treatments (ethanol ± OA). It has previously been determined that OA, a long chain free fatty acid, significantly induces LD formation in hepatocytes [28]. For fatty acid treatment to WIF-B cells, OA was conjugated with BSA (1.5%) in serum-free F-12 media prior to addition to the cell cultures. In general, confluent and polarized WIF-B cultures were treated with media (serum-free F-12 with 1.5% BSA) with and without OA (100 *μ*M–1000 *μ*M), 25–50 mM ethanol and/or 0.25 mM 4MP, an inhibitor of alcohol dehydrogenase. The cells were plated on coverslips and stained with BODIPY for microscopic analysis of LDs. In other cell cultures, LD formation in WIF-B cells was quantified following extraction and spectrophotometric detection of ORO from the stained culture dishes.

### 2.3. Triglyceride Extraction and Analysis

Extraction of triglycerides was performed using the Folch method [29] with slight modifications. Briefly, after exposure to 25 mM ethanol for 48 hours, WIF-B cells (approximately 3 × 10^6^ cells/60 mm dish) were rinsed with PBS, harvested by scraping, and the pellet reconstituted in PBS. An aliquot was saved for protein/DNA determination with the remaining extracted with the addition of chloroform/methanol (2 : 1) followed by vortexing for 20–30 seconds. The sample was filtered over Whatman number 1 filter paper with a further rinse with 1 mL chloroform/methanol. The final volume of chloroform/methanol was recorded. Aliquots (1 mL) were made and the samples were dried completely using a Centrivac. Following drying, the triglycerides were hydrolyzed by the addition of 95% Ethanol and 8.0 M KOH at 65°C for 20 minutes. The triglyceride content was determined using Triglyceride Reagent (Thermo Scientific) as directed by the manufacturer with detection made by spectrophotometric analysis (Beckman DU640).

### 2.4. Oil Red O Staining

ORO staining was performed as previously described [30] with minor modifications. In brief, WIF-B cultures were fixed in 10% buffered formalin, incubated with 60% isopropanol, and stained of 10 minutes with ORO solution (0.21% dye in 100% isopropanol). Following staining, the cultures were washed five times with sterile water, the dye is extracted using isopropanol, and the concentration of ORO in the extract was measured colorimetrically (500 nm). Results were expressed as OD/mg protein or DNA. In several experiments, images of ORO stained cells were obtained prior to extraction using an Olympus IX70 microscope in combination with a MicroFire digital camera (Image Processing Solutions, North Reading, MA) at ×100 and ×200 magnifications.

### 2.5. Oil Red O-Based Quantification of Fat Efflux

For rapid and convenient quantification of fat efflux from treated cells, WIF-B cultures were rinsed twice (PBS) and the media changed to oleate-free F-12 media with or without 25 mM ethanol. The plates were sealed and allowed to incubate for an additional 24 hr followed by staining with ORO for quantification of LD accumulation. The efflux of fat from the cells was determined by comparing the amount of ORO taken up by the cells before and after removal of oleate from the media.

### 2.6. Fluorescence Microscopy

To measure LD content by BODIPY staining, WIF-B cells that were cultured on glass coverslips were subjected to the various treatments, fixed for 20 min with formaldehyde and briefly (2 min) permeabilized with D-PBS + 0.1% Triton X-100. Following incubation with 5 *μ*g/mL BODIPY 493/503 in PBS, the coverslips were washed and mounted on glass slides using DAPI-containing UltraCruz mounting medium. Cells were viewed with a Nikon ECLIPSE 80i Microscope equipped with a Nikon DS-Qi1Mc digital camera (Boyce Scientific, Inc., Gray Summit, MO). Images were processed using NIS-Elements Imaging Software.

### 2.7. Western Blot Analysis

Cell protein was obtained from WIF-B cells by homogenization in 0.25 M sucrose in 5 mM Tris-HCl, pH 7.5 containing protease inhibitor cocktail (Sigma, St. Louis, MO). Cell protein was resolved on 12% reduced gels by SDS-PAGE and transferred onto nitrocellulose membranes. The blots were blocked for 1 hr in Odyssey blocking buffer (LI-COR Biosciences, Lincoln, NE) at room temperature and subsequently probed overnight at 4°C with primary antibodies, mouse antiadipophilin/ADRP (Fitzgerald, Acton, MA) at 1/500 dilution, and rabbit anti-rat GAPDH (Santa Cruz, Santa Cruz, CA) at a 1/5000 dilution. The blots were then incubated with secondary antibodies (IRDye680 goat anti-rabbit IgG and IRDye 800CW goat anti-mouse IgG) (LI-COR Biosciences, Lincoln, NE) at 1/10,000 dilution. Following washing, the blots were scanned and quantified using the Odyssey Infrared Imager (LI-COR Biosciences, Lincoln, NE).

### 2.8. Real-Time Polymerase Chain Reaction (PCR)

RNA was isolated from the WIF-B cells using a PureLink RNA Mini Kit (Invitrogen, Carlsbad, CA) according to the manufacturer's instructions. The concentration and quality (260/280 ratio) of the RNA was determined by a NanoDrop Spectrophotometer (NanaoDrop Technologies, Wilmington, DE). Real-time PCR reactions were performed using Tagman gene expression assay for rat adipophilin (Cat number RN01472318_m1) and rat actin (Cat number 4352931E) purchased from Applied Biosystems, Carlsbad, CA. Detection was performed using a 7500 Real Time PCR System (Applied Biosystems). The delta-delta Ct method was used to determine the fold change using actin for normalization.

### 2.9. Statistical Analysis

Results are expressed as mean ± SEM. Comparison of paired values was performed using the Students *t*- test with values *P* < 0.05 being considered significant. Comparisons among groups of data were made using one-way ANOVA with Tukey's post hoc test; *P* < 0.05 was considered significant.

## 3. Results

### 3.1. Effects of Fatty Acids and Ethanol on Lipid Accumulation in WIF-B Cells

**Figure 1:**
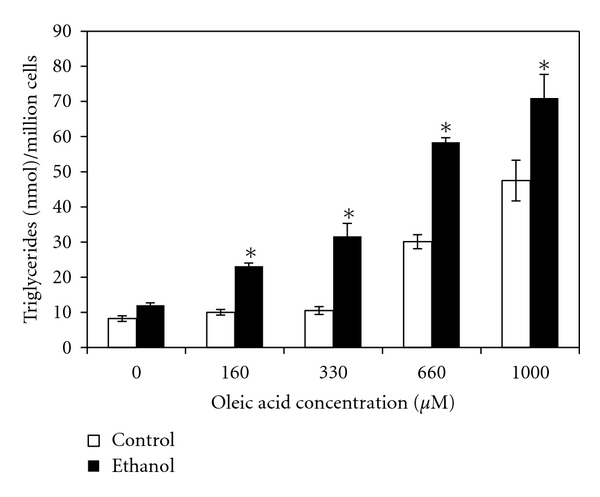
Ethanol-induced triglyceride accumulation in oleate-treated WIF-B cells. Cells were treated for 48 hr with or without 25 mM ethanol in presence of different concentrations of oleic acid. Values are means ± SEM (*N* = 4). *Significantly different (*P* < 0.05) from control group.

**Figure 2:**
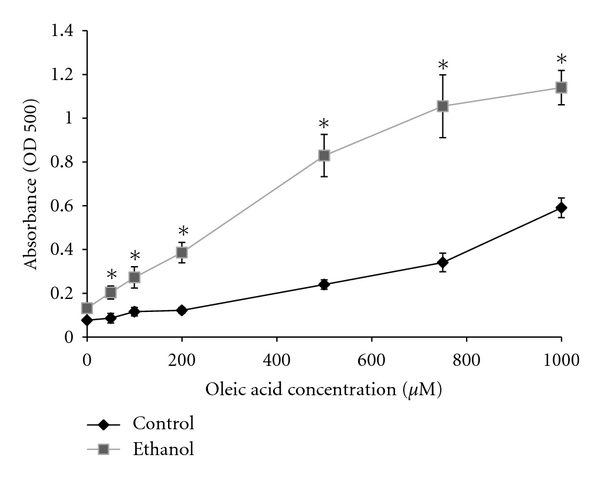
Oil Red O-based quantification of ethanol and oleic acid-induced fat accumulation in hepatoma hybrid cells. WIF-B** c**ells were treated for 48 hr with or without 25 mM ethanol in presence of different concentrations of oleic acid and lipid content quantified with Oil Red staining. Values are means ± SEM (*N* = 4). *Significantly different (*P* < 0.05) from control group.

**Figure 3:**
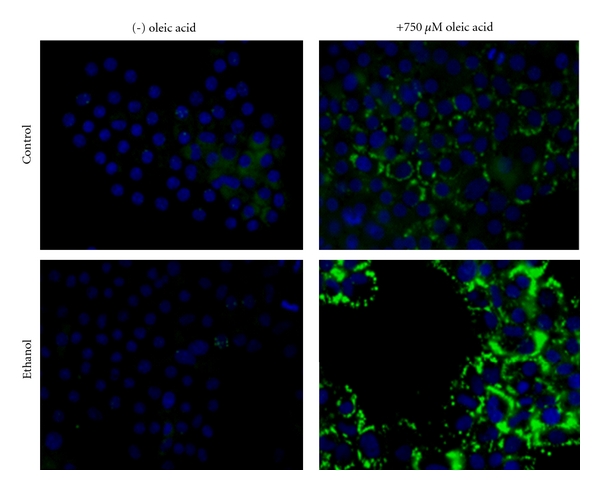
Lipid droplet accumulation in ethanol and oleic acid-treated cultures. WIF-B cells were treated without (control) or with 25 mM ethanol in the presence of 750 uM oleic acid followed by immunohistochemical analysis of the presence of lipid droplets by BODIPY staining.

It is known that alcoholic fatty liver is an early consequence of alcohol consumption. Central to this condition is the accumulation of lipids such as cholesterol esters and triglycerides that are packaged in lipid droplet (LD) organelles in hepatocytes [31–33]. However, the role ethanol metabolism has in steatosis and LD dynamics remains to be clarified. Here we analyzed fat accumulation in ethanol-metabolizing hepatoma hybrid (WIF-B) cells to better define steatosis at the cellular level. Initially, we investigated what effect ethanol treatment with or without the addition of exogenous free fatty acids would have on hepatocellular triglyceride levels. Specifically, WIF-B cultures were treated up to 48 hours with ethanol and/or oleic acid (OA), a monosaturated omega-9 fatty acid that has previously been identified as having a role in hepatic steatosis [28]. As expected, cellular triglyceride levels were found to be increased in a dose-dependent manner with OA treatment (Figure 1). Also, the addition of ethanol into the cell cultures resulted in a 2- to 3-fold enhancement in triglyceride levels over the concentration range of OA treatment (Figure 1). This noted enhancement of cellular triglycerides in the ethanol and OA-treated cells correlated with the accumulation of neutral lipids packaged into cytoplasmic LDs. This was demonstrated using an ORO-based colorimetric quantitative assay which detected the concentration-dependent elevations in vesicular lipid content following oleate treatment that doubled in the presence of ethanol (Figure 2). Microscopic analysis subsequent to BODIPY staining of the cytoplasmic LDs paralleled the observed OA and ethanol-induced increases in triglyceride and ORO-stained lipids (Figure 3).

### 3.2. Enhanced Fat Accumulation in WIF-B Cultures Requires Ethanol Metabolism

**Figure 4:**
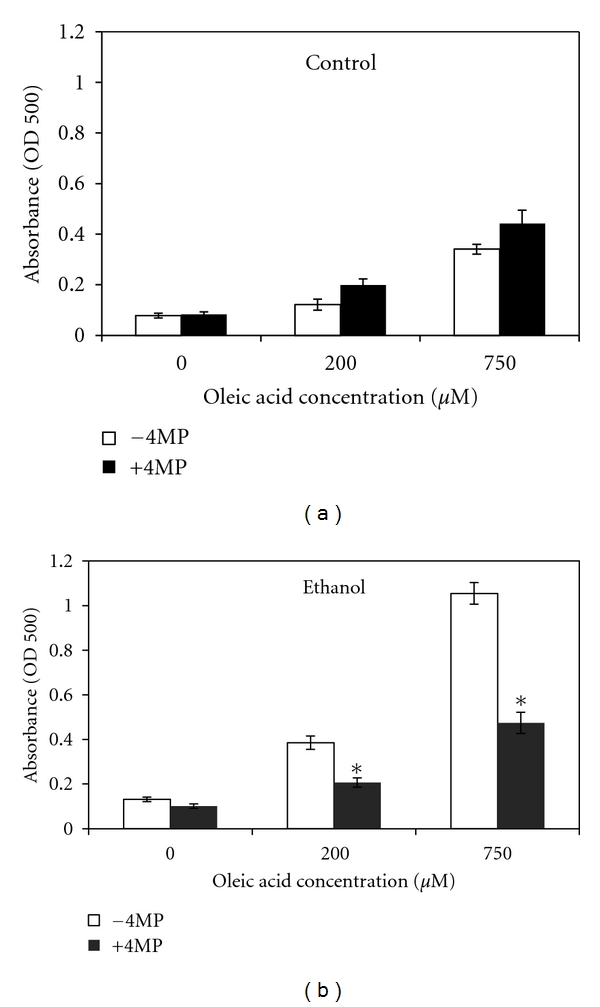
Ethanol and oleic acid induced fat accumulation requires ethanol metabolism. Oil Red O staining after 48 hours in the presence of increasing concentrations of oleic acid either with or without the addition of 0.25 mM 4-methylpyrazole (4MP) to inhibit ethanol metabolism. Results were from the ORO-based detection of neutral lipids in (a) oleate alone-treated (control) cells and (b) WIF-B cultures treated with both oleic acid and 25 mM ethanol. Values are means ± SEM (*N* = 4). *Significantly different (*P* < 0.05) from control group.

The role ethanol metabolism by alcohol dehydrogenase (ADH) has in the formation of LDs was tested by including a specific ADH inhibitor, 4-methyl pyrazole (4-MP) in the OA/ethanol-treated cultures. In WIF-B cells treated with oleic acid alone (control cells), the presence of 4-MP had no effect on cellular levels of neutral lipids detected by ORO staining (Figure 4(a)). However, in ethanol-treated cells, the inclusion of 4-MP reduced the accumulation of neutral lipids to control levels (Figure 4(b)). These results demonstrate that the metabolism of ethanol and the subsequent formation of reactive metabolites is associated with lipid droplet accumulation in the hepatoma cells.

### 3.3. Ethanol-Induced Effects Associated with Cellular Lipid Retention Include Alterations in Lipolysis, Lipid Efflux, and Cell Survival Mechanisms

**Figure 5:**
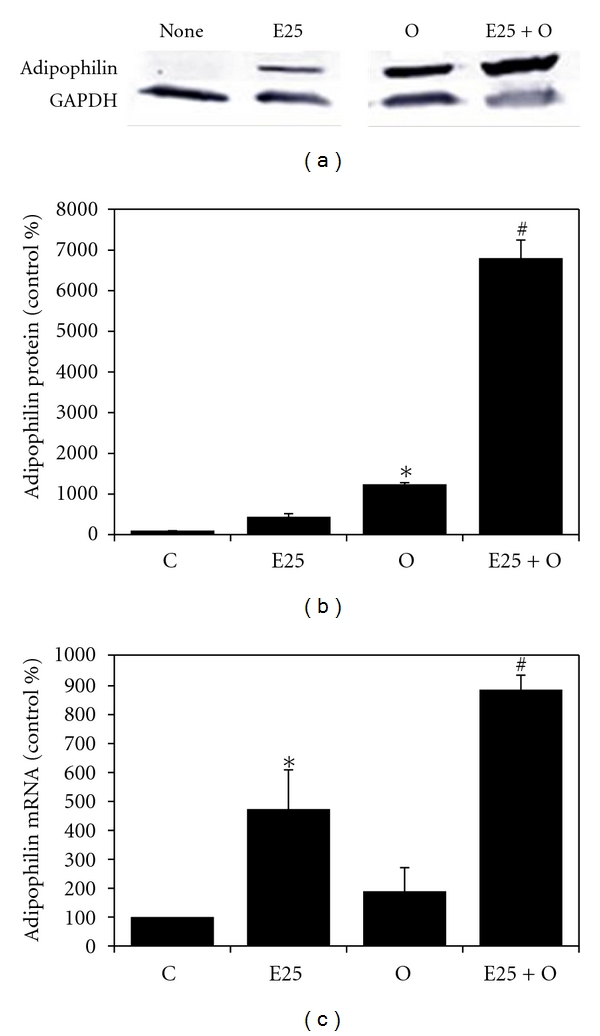
Adipophilin (ADRP) protein content and mRNA expression in WIF-B cells. WIF-B cells were cultured for 48 hours in the absence (control media, C) or presence of 25 mM ethanol (E25) with or without the presence of 0.5 mM oleic acid (O and E25 + O). (a) Representative Western blot indicating the presence of ADRP and glyceraldehyde-3-phosphate dehydrogenase (GAPDH) as the loading control. (b) Relative content of adipophilin protein expressed as percent of control from four independent experiments. (c) Adipophilin mRNA expression detected in the WIF-B cells. *Significantly different (*P* < 0.05) from control group and E25 group. ^#^Significantly different from all other groups (C, E25, and O).

**Figure 6:**
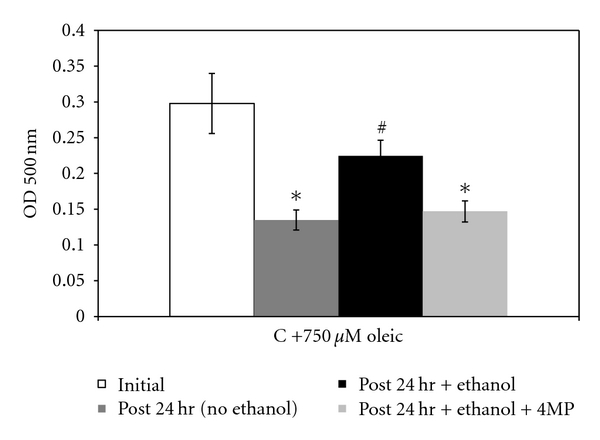
Fat efflux from hepatic cells is impaired by ethanol treatment. WIF-B cell cultures were “loaded” with fat for 48 hours, washed, and then reintroduced into fresh media. Oil Red O staining was measured before and after a 24-hour “washout” period. Data are expressed as fat content pre- and post-washout as intensities of Oil Red O for quantification. Values are means ± SEM (*N* = 4). *Significantly different (*P* < 0.05) from control (initial fat load) group. ^#^Significantly different from all other groups.

**Figure 7:**
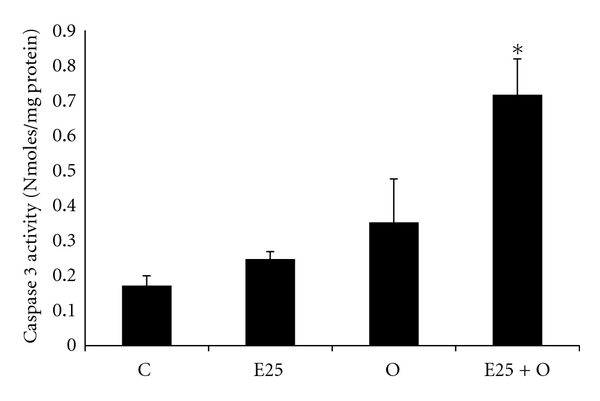
Ethanol and oleate-induced apoptosis in polarized hepatoma cultures. WIF-B cells were cultured for 48 hours in the absence (control media, C) or presence of 25 mM ethanol (E25) with or without the presence of 0.5 mM oleic acid (O and E25 + O). Apoptotic cell death was measured by spectrofluorometric detection of caspase-3 activity. Results are expressed as nanomoles of detected fluorometric substrate (AMC) cleaved and released by active caspase-3 enzyme per mg protein for four independent experiments. *Significantly different (*P* < 0.05) from control group.

As just described, the combined effects of ethanol and OA to WIF-B cells result in the substantial retention of lipids in the form of LDs. It is predicted that the ethanol-mediated lipid retention is, in part, due to impaired degradation of the accumulating LDs. In support of this prediction, we found that the combined treatment of ethanol and OA significantly elevated the expression of a LD-associated protein, adipophilin (Figure 5). Adipophilin, otherwise known as adipose differentiation-related protein (ADRP), is a well-characterized LD protein that is known to be involved in LD homeostasis particularly by playing a role in the attenuation of lipolysis [5, 34]. In addition to alterations observed in lipolytic mechanisms, it was also observed that ethanol and OA treatment resulted in a significant reduction in lipid efflux from the cells. The data in Figure 6 reflect this finding as significantly less of the accumulated lipid was released from ethanol-treated cells following starvation compared to those treated with OA alone. And finally, to determine if ethanol-induced steatosis could result in cell injury, we measured apoptosis in the ethanol- and OA-treated WIF-B cells. It was determined that the induction of hepatocellular apoptosis correlated the observed increase in LD accumulation. Specifically, in OA- and ethanol-treated cells where we observed the highest increase in LD accumulation, the activity of a key executioner enzyme of programmed cell death mechanisms (caspase-3) was significantly enhanced (2 to 3-fold, *P* < 0.05) in the presence of ethanol compared to OA alone-treated cells (Figure 7).

## 4. Discussion

The accumulation of lipids in the liver is an early pathological stage in the development of alcoholic liver disease (ALD) that occurs in most individuals that chronically consume alcohol [35, 36]. Furthermore, ethanol-induced fatty infiltration has been suggested to sensitize the liver to damaging risk factors such as oxidative stress and prodeath signaling mechanisms. However, the mechanisms involved in fatty liver disease represented as hepatocellular steatosis, and particularly the accumulation and/or regulation of lipid droplets (LDs) in hepatocytes, remains to be elucidated.

It is known that the accumulation of excessive lipid in hepatocytes can be related to alterations in mechanisms involving the uptake, synthesis, and esterification of free fatty acids [1, 37]. Also, the damaging effects of ethanol have been implicated in impairments in lipid degradation (lipolysis) as well as secretory mechanisms. Indeed, ethanol consumption has been linked to altered triglyceride and phospholipid synthesis, impairments in fatty acid oxidation, and the secretion of very low-density lipoproteins (VLDLs) [12–14, 38]. However, details remain to be determined concerning LD formation, accumulation, and lipolysis in ethanol-damaged hepatocytes. To date work has been completed describing LD accumulation in human and animal models along with the role of ADRP in LD maturation [39–41]. Also, a recent study has defined specific ethanol-mediated alterations in LD protein properties that are related to LD formation in steatosis [42]. Here, we contribute to the study of ethanol-induced LD accumulation by demonstrating that as a consequence of ethanol metabolism in polarized hepatoma cells, LD enhancement is related to changes in lipid efflux and ultimately cell survival. Importantly, these effects were demonstrated using WIF-B cells which are a well-characterized model to study hepatocyte protein trafficking machinery as well as the biological basis of ethanol-induced fatty liver. The WIF-B cells are natural ethanol-metabolizing cells and have been shown to accumulate triglycerides when exposed to ethanol in a manner similar to that observed in ethanol-fed animals. Thus, the WIF-B cells are an ideal model system to decipher consequences of ethanol-mediated enhancements in cellular triglyceride levels and related storage in lipid droplet organelles. Indeed, we observed a dose-dependent increase in LD accumulation in ethanol- and oleic-acid-treated WIF-B cells shown by the quantitative assessment of cellular triglycerides and staining of neutral lipids. Additionally, this observed ethanol-induced lipid retention in WIF-B cells was found to be related to changes in LD protein dynamics and cellular lipid efflux. It is known that hepatocytes can take up long chain fatty acids such as oleic acid which can then be esterified to neutral lipids (cholesterol esters and triglycerides) and packaged into and stored as phospholipid-covered LD organelles [3]. Under normal or fasting conditions, hepatocytes effectively metabolize and degrade the stored LDs. However, under hepatocellular damaging conditions (ethanol exposure), the storage of fatty acids is enhanced leading to the accumulation of LDs. The increase in cytoplasmic LDs was thought to be the result of ethanol-induced alterations in lipid metabolism. The work presented here shows that enhanced triglycerides and LD formation are also a consequence of the ethanol metabolism in the cell that results in changes to LD properties. To aid in our understanding of this observed hepatocellular LD retention, future work using the WIF-B cell model may contribute to the correlation of ethanol-mediated changes to hepatocyte membrane trafficking processes and the attenuation of LD disassembly.

The study of LD biology is an emerging area of investigation fueled by the knowledge that the dysregulation of neutral lipid stores is linked to a variety of disease states including alcoholic liver disease as we have described here. It is also not surprising that in those disease states, cellular LDs are thought of as active organelles whose composition, biogenesis, trafficking, and storage/degradation have been found to be complex involving a varied proteome and genome. In this study, we reported the effect ethanol metabolism has on one of the abundant and well-studied LD proteins, ADRP. Because of the availability of antibodies to LD proteins and protocols to identify LDs (e.g., ORO-based assays and BODIPY staining), we were able to show that ADRP protein levels and hence LDs were significantly increased when treated with ethanol, oleic acid, or both. The increase in ADRP appears to be partially controlled at the level of transcription as ADRP mRNA expression was increased as well. Therefore, since ADRP is well known for its role in lipid homeostasis particularly by attenuating lipolysis, our data demonstrate that the ethanol-amplified LD accumulation in hepatic cells involves impairments in the breakdown of lipids as a consequence of alcohol metabolism. We were also able to show that the metabolism of ethanol was necessary for alcohol-mediated effects on fat efflux. Particularly, the efflux of lipid stores in oleate-loaded cells was found to be significantly decreased when the cells were starved in the presence of ethanol. Moreover, the ethanol-mediated reduction in lipid efflux was abrogated in the presence of the alcohol dehydrogenase inhibitor, 4-MP. And finally, we demonstrated that the ethanol-mediated enhanced presence of LD organelles may be involved in the promotion of hepatocyte toxicity as the lack of proper fatty acid mobilization/secretion may lead to cell death signaling. Indeed, a measure of hepatic apoptosis was found to be increased in WIF-B cultures that were treated with ethanol and oleic acid, a finding that correlated to the observed accumulation of LDs under the same conditions. Ongoing work is aimed at delineating contributing mechanisms involved in the observed LD accumulation associated with the metabolism of ethanol. In particular, it is of interest to determine the role of ethanol-mediated alterations in LD-vesicle trafficking machinery of the hepatocyte as it is likely that the regulation of LDs involves modulation of vesicle-based degradative processes.

In summary, our findings suggest that the metabolism of ethanol in hepatocytes is directly related to impaired lipid lipolysis and fat efflux that contribute to increased hepatic LD accumulation. Moreover, we have identified that hepatic cell death may be a potential consequence of LD dysregulation and accumulation due to ethanol's alterations in LD properties. In addition to yielding therapeutic leads for alcoholic liver disease, the knowledge gained here may possibly be extended to other diseases that involve lipid accumulation, including nonalcoholic fatty liver disease, atherosclerosis, diabetes, and cancer. These conditions affect millions of Americans and are major health concerns. Future work examining how ethanol impacts LD vesiculation, trafficking, and targeting within the hepatocyte will likely contribute to the study of LD biology and our understanding of fatty liver disease.

## Abbreviations

ALD: alcoholic liver disease
LDs: lipid droplets
WIF-B: hybrid of human fibroblast (***WI*** 38) and rat hepatoma (***F***ao) cells
4-MP: 4-methylpyrazole
ADRP: adipophilin
ADH: alcohol dehydrogenase
CYP2E: cytochrome P-450 2E1.
